# Obstruction of the Bowels

**Published:** 1898-05

**Authors:** T. F. Prewitt

**Affiliations:** Professor of Principles and Practice of Surgery, Missouri Medical College; Surgeon to St. John’s Hospital, etc., St. Louis, Mo.


### OBSTRUCTION OF THE BOWELS.

Presented to the Medical Association of Missouri, May 20, 1897.

BY T. F. PREWITT, M.D.

Professor of Principles and Practice of Surgery, Missouri Medical College; Surgeon to St. John's Hospital, etc., St. Louis, Mo.

"Obstruction of the bowels" is a technical phrase which implies a mechanical obstacle to the onflow of the intestinal contents. Its causes are various and the obstruction may be complete or partial, acute or chronic. It may occur at any portion of the intestinal tract from the duodenum to the rectum. It may be due to conditions that are congenital or may be acquired. But whatever may be the character of the obstruction the one striking fact dominates all considerations relating to treatment—it is a mechanical obstacle. A simple reference to the schedule of causes, revealed by postmortems, establishes this fact and demonstrates as well the utter uselessness of medical measures.

It is impossible here to more than outline the symptoms, diagnosis and treatment of obstruction of the bowels, acute and chronic. It is necessary to deal with these two groups separately.

In the classification of intestinal obstruction it is necessary to take into consideration both the pathologic anatomy and clinical aspects of the subject and study the relations of one to the other.

A careful collection of a large number of cases examined postmortem has shown that one of the following causes is responsible, with rare exceptions, for every case of obstruction with which we meet: 1, internal hernia; 2, twist (volvulus); 3, bands of some kind; 4, fecal impaction; 5, mechanical pressure of tumors; 6, stricture; 7, contractures due to matting together of intestinal coils from peritoneal and cancerous diseases; 8, intussusception; 9, gall stones; 10, enteroliths; 11, foreign bodies. A more practical classification, however, is that in which all cases are divided into acute and chronic.

A study of the pathology in connection with the clinical history reveals a striking relationship between the causes and the group of symptoms which are associated with them. Thus, of the causes leading to acute obstruction we have: 1, internal hernia; 2, twists (volvulus); 3, bands; 4, intussusception; 5, gall stones. Of the leading causes of chronic obstruction we have: 1, fecal impaction; 2, mechanical pressure of tumors; 3, stricture; 4, contractures; 5, intussusception, which may be and is sometimes chronic.

So constant is the relationship between primarily acute obstruction and the causes grouped above that we can say that any given case is due to one of these, and the same may be said of primarily chronic cases.

*Symptoms.*—In acute cases these come on suddenly in a previously healthy individual and are striking and characteristic.

The attack is sudden and acute. Pain is paroxysmal, central and fixed. Vomiting sets in early, first of the contents of the stomach, then of the duodenum, then of the small intestines and rapidly becomes fecal. The patient becomes faint, pale and markedly collapsed; constipation is absolute, not even flatus escaping from the bowels. Distention of the abdomen rapidly takes place and distended coils of intestines may often be both seen and felt. The urine may be scanty or even suppressed.

It will be seen that these are the symptoms of acute strangulated hernia. And why not? There is the same interruption to the onflow of the intestinal content; the same obstruction to the blood current; the same disturbing and depressing effect on the nervous system; the same violent peristalic action in the endeavor to overcome the obstruction and push on the obstructed contents; the same paroxysmal pains, and eventually the same fatal result from precisely the same causes.

When, therefore, we are confronted with a case presenting such a group of symptoms there can be no doubt as to the diagnosis. A dire disaster has befallen the patient. Death confronts him, and only the coolness, the courage and skill of the surgeon can rescue him from its grasp.

The bowels do not move. Shall we give purgatives? Already the bowel is making frantic efforts to force the blockade. Shall we stimulate it to more furious efforts to accomplish the impossible? No. It would be gross malpractice.

The patient suffers great pain. Shall we give opium? If it be used only as an adjuvant to annul pain, to relieve collapse, to restore the action of the kidneys, if it is administered for these purposes only while the surgeon prepares to relieve the strangulation, yes. If it is given with any curative purpose, then emphatically, no. We do but invite euthanasia. The patient relieved of his suffering is deluded with the idea that he is doing well; the doctor, no longer assailed by the cries of the patient, too often hesitates, and the golden moments slip by during which the patient might be rescued from an otherwise inevitable doom. When the diagnosis is once made every hour of delay is compromising his safety. The bowel is damaged, peritonitis sets in, collapse ensues and the patient dies in spite of the delayed operation.

When the diagnosis is made celiotomy is the only thing consistent with that condition. If there be any doubt an exploratory celiotomy should be done to remove that doubt.

It is scarcely worth the time to discuss certain measures that are advised in these cases. Of what use is irrigation or lavage of the stomach except for the purpose of putting the patient in a better condition for operation, or distention of the colon with fluids except for the same purpose? Tubage of the colon is a delusion; manual exploration of the rectum is a bit of brutal manipulation rarely jutifiable. Taxis and massage, though lauded by that master in surgery, Jonathan Hutchinson, is useless and dangerous. Compression of the abdomen is a waste of time. None of these are curative, and they but serve to divert the attention from the one thing that can relieve the difficulty.

I have been thus earnest in advocating early operation in these cases because I have seen them treated by the exhibition of the most powerful purgatives, by the administration of a quarter of a pound of liquid mercury, by delay extending over days, until the onset of peritonitis, the moist clammy skin, the hiccough, the stercoraceous vomiting, the quickened pulse, proclaim any operation useless.

A few words must be said in regard to the differential diagnosis and treatment of acute intussusception, for while it presents the usual symptoms of obstructions of the bowels, there are certain symptoms peculiar to this accident. By the term intussusception we mean the inversion or prolapse of a portion of the bowel into the lumen of the part immediately adjoining. No portion of the bowel is exempt from this accident, though the relative frequency varies in different portions of the bowel. In the inversion of the finger of a glove we have an exact representation of what takes place in intussusception, except that in the inversion of the bowel the mesentery is carried in with the intussusceptum.

The role played by the mesentery is important. As the invagination extends the traction upon it increases, the tumor of which it forms a part is rendered somewhat crescentic, the pressure on the lumen of the bowel is increased and facility of its reduction greatly interfered with. The most frequent variety is the ileocecal, the next the enteric, then the colic, while the ileocolic is the least frequent.

It is well to remember that intussusceptions occur in the dying. But as they give rise to no symptoms during life, indeed are discovered only postmortem, they do not concern the surgeon. The absence of all indications of congestion or inflammatory action and the facility with which they can be reduced render the diagnosis postmortem easy. In intussusception the constipation is not so absolute except in the very acute cases. A certain amount of diarrhea at the onset is frequent in a large proportion of the cases, constipation becoming complete toward the close of the case.

In consequence of the great engorgement of the invaginated portion of the bowel (the intussusceptum) a certain amount of blood is found in the stools. Tenesmus is a striking and usually an early symptom, and the occurrence of marked tenesmus with bloody mucus in connection with other symptoms of obstruction is almost pathognomomic. It is often mistaken for disentery. Vomiting is usually not so marked or distressing a symptom in intussusception. The presence of a tumor formed by the invaginated bowel can in nearly 50 per cent. of the cases be felt. It can more frequently be recognized in children than in adults and is somewhat sausage-shaped. It is most often to be felt over the transverse and descending colon. When it can be found it is a great aid in diagnosis.

In the ultra acute cases death follows within twenty-four to forty-eight hours; they are always fatal, in fact. Fortunately the ultra acute cases are rare.

As to the treatment, I have succeeded in relieving quite a number of cases by distending the lower bowel with air or gas. I believe that an early resort to this method, before adhesions between the different layers of peritoneum have taken place, will often prove successful. Failing in this, an early resort to celiotomy is demanded to reduce the invagination. This is much more readily accomplished before the great engorgement of the invaginated portion has taken place and peritonitis with adhesions of the peritonal folds has set in.

The reduction of the invagination is best accomplished by a process of pushing the intussusceptum out from below while slight traction is made from above. A direct pull on the invaginated portion of the bowel is apt to lead to tearing of its walls, and the danger is the greater the longer the invagination continues. Obstruction in some of its forms is by no means infrequent, nor is the diagnosis always easy. In fact, judging from the injudicious treatment adopted, we must believe that the true condition is not recognized or at least not appreciated in many cases. Postmortem investigations have thrown a flood of light on these cases, and every fairly-well posted practitioner should be familiar with their pathology.

While it would be desirable to make a differential diagnosis as to the clinical cause in every case, it can not be expected to do more than approximate the special cause in a given case. Nor should it influence the course to be pursued. The suddenness with which appendicitis develops, the severity of the pain, the tendency to collapse and the occasional vomiting might mislead the practitioner and lead to some confusion as to the diagnosis as between obstruction and that disease. But the consideration of a few points of difference ought to clear up the diagnosis

Appendicitis is an inflammatory affection *ab initio*. In obstruction, inflammation is a secondary affair.

The peritoneal irritation developed in appendicitis often causes great tenderness all over the abdomen, most marked, however, over the region of the appendix, and later receding until it is limited to that region.

Pressure over the abdomen in obstruction often adds to the comfort of the patient.

The pain in appendicitis is more continuous and localized. In obstruction it is central and paroxysmal. Vomiting in appendicitis is by no means constant. In obstruction it is rarely absent. In short, the arrest of peristalsis resulting from inflammatory conditions leading to a quasi obstruction has no place in this discussion.

Internal hernia must be reduced, volvulus must be untwisted, bands must be divided, etc.

Chronic obstruction of the bowels is marked by symptoms quite distinct from those of the acute. It will be found generally that there is a history of long standing trouble of the bowels, some colicky pains, difficulty of getting the bowels to move, and there may be blood or mucus in the stools and a change in the form and character of the passage. At one time diarrhea may be present, at another constipation, etc. These symptoms gradually increase in severity until the patient dies from exhaustion, or it may be acute symptoms supervene upon the chronic and the patient dies from collapse or peritonitis. The history is one of chronic obstruction, and though the patient may have, when seen, tympanitic distention, paroxysmal pain, visible peristalsis, vomiting, hiccough, etc., the canse will be found to be one of those I have enumerated, viz.: fecal impaction, pressure of a tumor, stricture of the bowel, or contractions.

The most frequent of these is stricture of the large or small intestine. About 60 per cent. of the cases of chronic obstruction from all causes examined postmortem have been found to be due to this cause, only 25 per cent. having been found in the small intestines, showing an immense preponderance in favor of the large bowel. In fact, it may be said that stricture is the cause of obstruction in the large bowel while "contractions" are the most invariable cause of obstruction of the small. Strictures are, with few exceptions, cancerous and epitheliol in character.

In a table of ninety-eight cases, compiled by Treves, the relative frequency of the different portions of the large bowel involved was as follows: Rectum and sigmoid flexure, 58; descending colon, 11; splenic flexure, 7; transverse colon, 7; hepotic flexure, 9; ascending colon, 2; cecum, 4.

The symptoms of stricture are alternating constipation and diarrhea, frequent mixture of blood and mucus with the stools and pain in defecation. When low down in the rectum the tumor can be felt with the finger, a means of diagnosis that should never be omitted. When in other portions of the colon it may often be felt through the abdominal parietes. The abdomen may be distended and, when distended, is most marked in the lumbar and epigastic region, in short, in the line of the large bowel. Large coils of the distended bowel with visible peristalsis are often seen, the result of the hypertrophy of the muscular coat, brought on by the increased peristalsis, extending over a long period of time, to overcome the resistance offered to the onflow of the intestinal contents.

The treatment of stricture of the colon depends somewhat on its location. If it be the rectum, extirpation of the entire growth at an early period is the remedy. If higher in the bowel, resection in favorable cases, and when this measure is impracticable, colotomy at a point above the stricture is indicated.

"Contractions," so named by Fagge, are a cause of obstruction in the small bowel and due to the matting together of the coils of the intestines from peritoneal and cancerous disease. In these cases the normal peristaltic action of the bowel is interfered with by the adhesions of the adjacent coils, by the bending and doubling of the bowel upon itself, thus interfering with the passage of its contents. This leads to the griping colicky pain often associated with the sick stomach coming on an hour or more after the indigestion of food and during the passage of the chyme through the obstructed portion of the bowel. Some distention may occur during the attack and, if so, is central and hypogastric; in other words, in the region occupied by the small intestines. The intestines will be seen writhing and coiling, a gurgling produced by the movement of the gases, and distinct peristaltic movements are perceived. With the escape of the contents through the obstructed portion of the bowel the patient is relieved of pain and discomfort until after the next meal, when the same symptoms recur. As there is no trouble with the large bowel the actions are normal and painless.

Something like 30 per cent. of the cases of chronic obstruction are due to this cause. The treatment must be plainly palliative, consisting in a careful regulation of the diet and the administration of mild laxatives. Should the symptoms become more urgent and the suffering great, enterotomy should be done and an artificial anus established.

As regards the obstruction caused by tumors, the treatment resolves itself into the treatment of the tumors themselves, though colotomy may be required in certain cases.

Last but not least in importance are those cases of obstruction due to the accumulation of feces. The importance of examining the rectum in all cases of obstruction of the bowels is emphasized in these cases. A source of error in the diagnosis in this condition is the fact that a quasi diarrhea may exist. While the bowel is loaded with fecal matter, a channel may be furrowed by the side of the mass, by which the thinner contents of the bowel higher up make their way and escape from the anus. It is not a rare thing to find that the practitioner has administered anodynes and astringents to control this supposed diarrhea.

The foundation of this condition is an habitual constipation brought on usually by sedentary habits, neglect of the individual to heed the desire to act, a gradual diminution of the reflex excitability of the spinal centers and such a stretching of the bowel that it fails to take cognizance of the presence of fecal matter.

This chronic constipation may lead to that condition known as "ileus paralyticus," in which a considerable portion of the bowel may become incapable of peristaltic action and absolute obstruction occurs. A mass of fecal matter accumulates in this section of the bowel and the portion above is incapable of exercising sufficient force to push it along. With time the accumulation becomes harder and harder, the bowel below has a tendency to contract and adds to the difficulty of emptying it. The continued accumulation of fecal matter distends the bowel still more until rupture may take place. Ulcerations are frequent and peritonitis may develop. Like all other forms of chronic obstruction the symptoms are likely to become aggravated and eventually to become acute.

The length of time during which the bowel may remain blocked is in some cases astounding. Dr. Thos. Strong reports a case in which no evacuation took place for eight and a half months. The extent to which the colon may become distended is very great. In a case which I saw some years ago, a prominent business man of this city, the whole colon from the cecum to the sigmoid flexure was filled and it could be felt as a large roll movable laterally, swinging upon the meso-colon, and evidently several inches in diameter. The presence of such a mass in the large bowel is a diagnostic feature of great value. In the descending colon and sigmoid flexure this accumulation may be divided into scybalous masses, forming what has been compared to a large rosary.

Would any one regard the use of purgatives as a rational method of treatment in these cases? I think not. It would not only be useless but as illogical as an attempt to force out a cork from a bottle by explosives from within that might more readily be extracted by a corkscrew.

The treatment of these cases should consist in the repeated and copious enemata of warm water, with or without the addition of other ingredients such as turpentine, soap, oil, etc. To be effective the enemata should be introduced slowly with the patient in the knee-chest position, so that the fluid may traverse the whole length of the colon. Massage may be resorted to advantageously as well.

Cases occur where none of these measures prove effective in breaking down and dislodging these fecal masses, and the symptoms of obstruction persist. The surgeon is then left the alternative of opening the abdomen and, by direct manipulation of the bowel, compressing and pushing on the accumulation to a portion of the intestine still capable of peristaltic action.

Should the existence of ulceration or peritonitis or other conditions forbid such a course, colotomy would be advisable, removing the fecal matter through the opening and subsequently stitching it up or establishing an artificial anus as might seem best.

In conclusion I would submit the following propositions:

1. Purgatives are absolutely contraindicated in all cases of acute obstruction, and are of very limited, exceptional and temporary advantage in chronic cases.

2. The administration of opium as a remedial agent is to be strongly condemned. It literally "smoothes the pathway to the grave," lulls to sleep and lures to death.

3. Obstructions of the bowels are strictly surgical and demand surgical measures for their relief.

NOTE.—In the preparation of this paper I have been indebted to various sources for valuable suggestions, especially to the essay of Mr. Frederick Treves of London on "Intestinal Obstruction."

—*The Journal.*

